# Color Image Generation from LiDAR Reflection Data by Using Selected Connection UNET

**DOI:** 10.3390/s20123387

**Published:** 2020-06-15

**Authors:** Hyun-Koo Kim, Kook-Yeol Yoo, Ho-Youl Jung

**Affiliations:** Department of Information and Communication Engineering, Yeungnam University, Gyeongsan 38544, Korea; kim-hk@ynu.ac.kr (H.-K.K.); kyoo@ynu.ac.kr (K.-Y.Y.)

**Keywords:** artificial intelligence, heterogeneous transfer method, image generation, LiDAR sensor, LiDAR imaging, learning systems, selected-connection network, sparse input data

## Abstract

In this paper, a modified encoder-decoder structured fully convolutional network (ED-FCN) is proposed to generate the camera-like color image from the light detection and ranging (LiDAR) reflection image. Previously, we showed the possibility to generate a color image from a heterogeneous source using the asymmetric ED-FCN. In addition, modified ED-FCNs, i.e., UNET and selected connection UNET (SC-UNET), have been successfully applied to the biomedical image segmentation and concealed-object detection for military purposes, respectively. In this paper, we apply the SC-UNET to generate a color image from a heterogeneous image. Various connections between encoder and decoder are analyzed. The LiDAR reflection image has only 5.28% valid values, i.e., its data are extremely sparse. The severe sparseness of the reflection image limits the generation performance when the UNET is applied directly to this heterogeneous image generation. In this paper, we present a methodology of network connection in SC-UNET that considers the sparseness of each level in the encoder network and the similarity between the same levels of encoder and decoder networks. The simulation results show that the proposed SC-UNET with the connection between encoder and decoder at two lowest levels yields improvements of 3.87 dB and 0.17 in peak signal-to-noise ratio and structural similarity, respectively, over the conventional asymmetric ED-FCN. The methodology presented in this paper would be a powerful tool for generating data from heterogeneous sources.

## 1. Introduction

In general, the light detection and ranging (LiDAR) sensor emits laser light and receives reflected light [1,2,3,4,5,6,7,8,9,10,11,12]. The reflected light conveys the distance to the target objects and the reflectivity of their surfaces. This intrinsic operational principle makes the LiDAR data independent of changes in the ambient illumination, unlike camera images. Because it provides consistent data regardless of time of day, the sensor has been used for various applications, such as object recognition [1,2,3] in driving environments, 3D roadmap construction [4,5], and semantic segmentation [6,7,8,9,13,14], etc.

**Figure 1:**
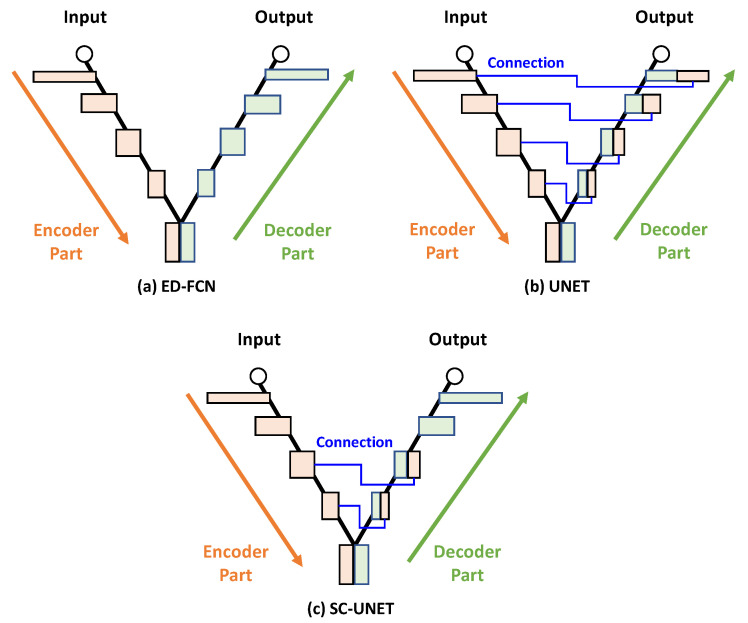
Various network architectures for semantic segmentation.

There have been recent studies on generating camera-like images from LiDAR data [10,12]. The LiDAR to color image generation is useful in various applications such as vehicle’s night vision system, night surveillance sensor, and military night vision device, etc. An encoder–decoder structured fully convolutional network [15] (ED-FCN) is used for image generation from the heterogeneous data in [10,12], as shown in Figure 1a. One interesting result discussed in [10,12] is that the shadow-free images are generated since the LiDAR reflection data are produced irrespective to the illumination change. This would be very useful property for visual assistance in night driving. The monochrome images can be generated from the LiDAR reflection data by using the ED-FCN [10]. An asymmetric ED-FCN architecture is proposed to generate color images from the LiDAR reflection data [12] in which the deeper decoder network than encoder is used. The asymmetric ED-FCN outperforms two conventional interpolation methods, such as nearest neighbor [16] and inverse distance weighted [17], and generative adversarial networks (GANs) based colorization method [18]. The GAN-based generation has peculiar phenomenons that the existing and non-existing objects are intermittently disappeared and appeared, respectively, and that the object locations are changed in the generated image [18].

Originally, the ED-FCN has been developed for semantic segmentation [6,7,8,15,19,20,21] and classification [3]. In the case of the modified ED-FCN, called UNET as shown in Figure 1b, the feature maps of the encoder network are combined into the maps of the decoder network via concatenation for bio-medical image segmentation [22]. The UNET is also used for the semantic segmentation for the LiDAR reflection data [9]. Recently selected connection UNET (SC-UNET), shown in Figure 1c, is proposed to detect the concealed object in the THz image for military purposes [23], resulting in additional improvement over UNET. Modified UNET and ED-FCN, i.e., UNET++ and RTFNet, are proposed for medical and urban scene semantic segmentation, respectively [13,14]. To the best of our knowledge, the UNET and SC-UNET have not been used for color image generation from LiDAR reflection data in the literature.

In this paper, we propose to use the SC-UNET structures for the camera-like color image generation from LiDAR reflection data. It should be noted that the input refection data are extremely sparse while the output image is dense. This difference in the sparseness yields that feature maps in the encoder and decoder have different characteristics in terms of sparseness and similarity. The differences in feature map characteristics are also varied with respect to the levels due to the network structure. In this paper, the sparseness of feature maps is analyzed based on receptive fields for each level in the ED-FCN network. In addition, the similarities between feature maps of the encoder and decoder are empirically analyzed by using the dataset recorded under various driving environments. Based on these analyses, we propose a methodology in selecting connections in the SC-UNET-based image-generation network. The connections between feature maps of the encoder and decoder parts in the proposed network are determined by considering the sparseness of each level in the encoder network and the similarity between the same levels of encoder and decoder parts.

The rest of this paper is organized as follows. In Section 2, we propose a network structure to generate a camera-like 2D color image from the 3D LiDAR data. The training and inference processes are also described. In Section 3, the performance of the proposed network is compared with the conventional ED-FCN and UNET networks. Section 4 draws the conclusions.

## 2. Proposed Method

In this section, we propose an image-generation network that generates a color image from the heterogeneous LiDAR reflection intensity. First, ED-FCN-based image-generation system [10,12] is analyzed with respect to sparseness and similarity. Then, the conventional SC-UNET architectures used for terahertz image segmentation [23] is re-purposed and adapted to heterogenous image generation based on the analyses.

### 2.1. Sparseness and Similarity of ED-FCN

**Figure 2:**
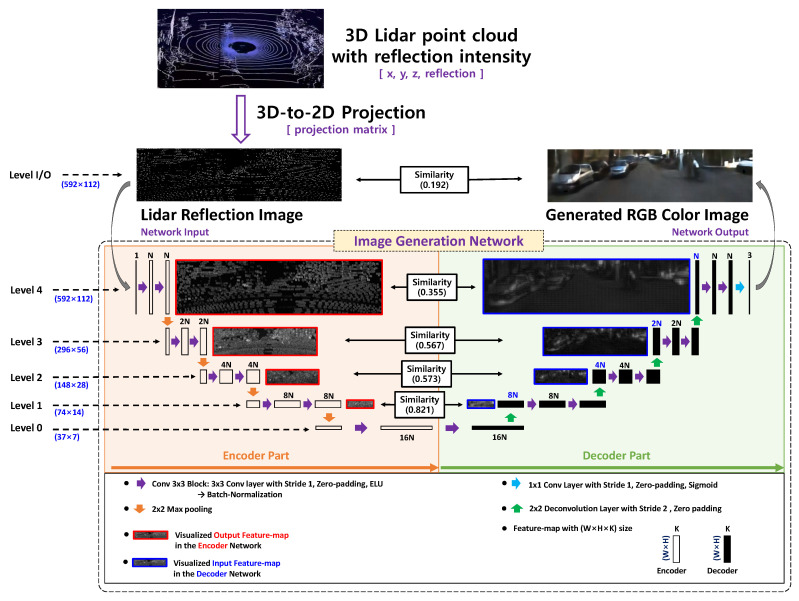
Encoder-decoder structured fully convolutional network (ED-FCN)-based color image-generation network from light detection and ranging (LiDAR) reflection data; the network has five levels including transition level (level 0); for each level, the similarities between representative feature maps of encoder and decoder parts are provided; the kernel size of the convolution filter is $3\times 3\times C_{L}$, where $C_{L}=2^{\left(4-L\right)}\times N$ represents the number of channels at the level *L*.

Figure 2 shows the ED-FCN-based image-generation system proposed in our previous works [10,12] and its feature maps at each level. In the pre-processing stage, 3D LiDAR point clouds are converted into a 2D LiDAR reflection-intensity image using a 3D-to-2D projection matrix. The reflection image has the same spatial resolution as the RGB color image to be generated. The color image is finally generated from the reflection image using the ED-FCN that consists of five levels with two convolution blocks. At each level of both the encoder and decoder blocks, $C_{L}\left(=2^{\left(4-L\right)}N\right)$ feature maps, denoted as $F_{L}^{e}$ and $F_{L}^{d}$, are obtained, where *L* and *N* indicate level number and filter number of the convolutional block at level 4, respectively. The dimension of the feature maps and the kernel size of the convolution filter are $W_{L}\times H_{L}\times C_{L}$ and 3 × 3 $\times C_{L}$, respectively. Two feature maps from encoder and decoder parts are visualized with representative feature maps, $R_{L}^{e}$ and $R_{L}^{d}$, respectively, in which each pixel is represented by the maximum value of the feature maps as follows:

(1) $$R_{L}^{e}\left(w,h\right)=\max_{c\in C_{L}}\left\{F_{L}^{e}\left(w,h,c\right)\right\}$$

(2) $$R_{L}^{d}\left(w,h\right)=\max_{c\in C_{L}}\left\{F_{L}^{d}\left(w,h,c\right)\right\}$$

The input reflection image is extremely sparse, i.e., the sparseness is 94.72%. This means that only 5.28% of the pixels in the reflection image have non-zero valid values and are irregularly distributed. In the encoder, the sparseness of the feature map is decreased as the level approaches the transition between the encoder and decoder parts, i.e., level 0. The feature map at the transition is completely dense (sparseness 0%). This is caused by enlarging the receptive field through a series of convolution and pooling processes. On the other hand, all the feature maps of the decoder part are dense. Detailed analysis of the relationship between the receptive field and sparseness is presented in Appendix A. If the UNET structure is directly applied to image-generation network, the sparse feature map in the encoder is combined with the dense one in the decoder at a higher level. For example, given that the encoder feature map has n% non-zero values, $\frac{100-n}{200}\%$ of a concatenated feature map is invalid and has an undesirable effect on generating the next feature map in the decoder. If the influence of the activation function is neglected, the percentage of non-zero values (n%) at each encoder level can be estimated by calculating the size of the receptive field. Accordingly, it is reasonable to apply the SC-UNET architecture, which concatenates feature maps at the levels at which the sparseness is lower than a certain value.

As shown in Figure 2, the input reflection intensity and output color images have completely different visual characteristics. The encoder and decoder feature maps at higher levels have characteristics similar to those of the reflection intensity and camera image, respectively. On the contrary, the feature maps of the encoder and the decoder have more common characteristics for a lower level. To verify the properties, the similarity $S_{L}$ [24,25] between representative feature maps at the level *L* is measured as follows:

(3) $$S_{L}=\frac{<R_{L}^{e},R_{L}^{d}>}{∥R_{L}^{e}{∥}_{2}{∥R_{L}^{d}∥}_{2}}$$

where <,> and ${∥\cdot ∥}_{2}$ denote inner product and L2 norm, respectively.

As shown in Figure 2, the similarity increases as the spatial resolution of the feature map decreases. For example, the similarity between input reflection and output color images is very low, i.e., 0.192. However, the similarity at level 1 is quite high, i.e., 0.821. Clearly, it is reasonable to concatenate feature maps with high similarity.

From the above analysis, the sparseness of the encoder feature map and the similarity between the encoder and decoder feature maps should be considered when designing the concatenation structure in an image-generation network.

### 2.2. Proposed Network Architectures

**Figure 3:**
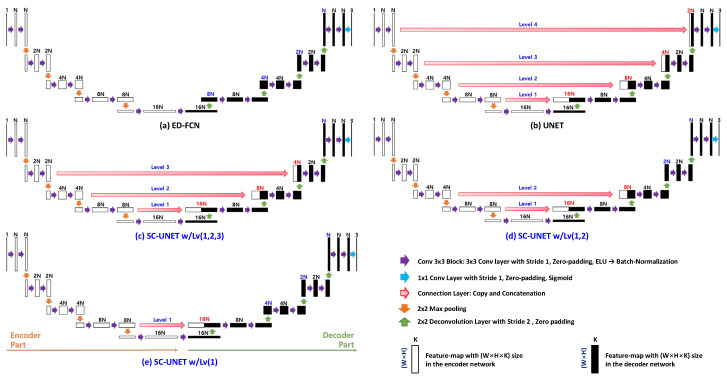
Conventional and proposed selected connection UNET (SC-UNET) network architectures for color image generation from LiDAR reflection intensity. (**a**,**b**) Conventional network architectures; (**c**–**e**) proposed SC-UNET network architectures; the feature maps of the encoder part are combined in the form of concatenation into the feature maps of the decoder part in the networks shown in (**b**–**e**).

In this section, we present the five types of network architecture for color image generation, as shown in Figure 3. ED-FCN represents the conventional architecture without any connection. UNET is also a conventional architecture that has feature map connections between the encoder and decoder parts at every level. The proposed architectures are the image-generation networks based on SC-UNET structures and are denoted as SC-UNET w/Lv(*a*,*b*,*c*), which indicates the SC-UNET architecture with the connection between encoder and decoder at levels *a*, *b* and *c*. Note that UNET is SC-UNET w/Lv(1,2,3,4).

All architectures consist of fully convolutional networks and have the following common structure. A single-channel sparse 2D reflection-intensity image (592 × 112 × 1) is obtained from the 3D LiDAR point and is used as input data to the image-generation network. The output of the generation network is a three-channel color image (592 × 112 × 3). The encoder and decoder parts of the network are constructed with five levels considering the size of the input image. Each level consists of two convolution blocks and one sampling layer. Each convolution block is composed of a convolution layer, exponential linear unit (ELU) activation function [26], and batch-normalization layer [27], in consecutive order. Each convolution layer consists of $2^{\left(4-L\right)}N$ filters of size 3 × 3, as shown Figure 3. In each convolution layer, stride 1 and zero-padding are applied. In the encoder, max pooling with factor 2 is applied for downsampling. In the decoder part, deconvolution [28] with stride 2 and zero-padding is applied for upsampling. As the level number of the encoder is decreased by one, the number of feature map channels is doubled. When the level number of the decoder is increased by one, the number of feature map channels is halved. At the end of the decoder part, the *N*-channel feature map is transformed into three color channels (*R*, *G*, *B*) by applying three 1 × 1 ×N-sized convolution layers with sigmoid activation, $s\left(x\right)=1/\left(1+e^{-x}\right)$. Notably, batch normalization is not applied for the 1 × 1 ×N convolution layers.

Conventional UNET and three proposed architectures have connections between the encoder and the decoder parts, unlike the ED-FCN. The encoder feature map at a certain level are connected to the decoder feature map at the same level in the form of concatenation. The concatenated feature map is fed to the convolution block of the decoder part.

The results of the analysis in Section 2.1 and the number of weights in the encoder feature map at each level are summarized in Table 1. The following observations are derived:

**Observation** **1.** *At a low level, the small amount of valid information can be transferred to the decoder side via concatenation. For example, the amount of feature map data to be transferred is very limited if only level 1 is concatenated.*

**Observation** **2.** *At a high level, the encoder feature map has high sparseness. For example, the structure having a single connection at level 4 is expected to have limited performance due to the small number of valid pixels.*

**Observation** **3.** *At a low level, the similarity between feature maps of the encoder and decoder parts increases. For example, the structure with a single connection at level 4 is expected to have limited performance due to the very different characteristics between the encoder and decoder feature maps.*

In summary, it is necessary to concatenate multiple levels in the sense of the amount of transferred information and it is desirable to concatenate feature maps at the low levels. Accordingly, we propose architectures, called SC-UNETs with w/Lv(1), w/Lv(1,2) and w/Lv(1,2,3).

### 2.3. Training and Inference Processes

In the training process, the 2D LiDAR reflection intensity images and the corresponding RGB color images are used as input data and target data of image generation network, respectively. Because the sigmoid function [29] is used as the activation function of the last convolution layer, the dynamic range of generated output data is (0,1). Thus, the target color images are converted to the same dynamic range for the training. Like as in [10,12], mean square error (MSE) is used as a loss function.

For hyper-parameters of training, the proposed network architectures are trained until a maximum of 2000 epochs. The adaptive moment estimation solver [30], with batch size 4, learning rate $l_{r}=5\times 10^{-4}$, and momentum parameters $\beta_{1}$ = 0.9, $\beta_{2}$ = 0.999 and ϵ = $10^{-8}$ is applied. The early stopping technique with patience parameter of 25 is applied for validation loss [31].

In the inference process, three-channel images with the dynamic range (0,1) are generated through the proposed color image-generation network. Finally, RGB color images are obtained by converting each channel to the dynamic range of (0,255).

## 3. Simulation Environment and Results

This section describes the simulation environments and evaluation metrics. The performance of the proposed architectures is evaluated and compared with the conventional architectures.

### 3.1. Simulation Environment

The evaluation dataset was reconstituted from the raw KITTI dataset [32], as in [10,12]. The dataset consisted of pairs of projected LiDAR reflection images and color images that were recorded simultaneously. Pairs recorded under heavy shadows were not included to enable shadow-free color image generation. For more details on the dataset, refer to [10,12]. The evaluation dataset consisted of a total of 4300 pairs. The pairs were randomly selected and divided into five folds for *k*-fold cross validation (k=5) [33,34]. Both LiDAR reflection and color images had the same resolution of 592 × 112 (66,304 pixels). The reflection image had an average of 3502 valid values.

The peak signal-to-noise ratio (PSNR) and structural similarity (SSIM) were used to evaluate the image quality between the generated and target color images [35]. PSNRs were separately calculated for each *R*, *G*, and *B* channel and the average PSNR was used for evaluation. In contrast, only the gray-scale image was used for the measurement of SSIM.

The hardware used in the simulation was a workstation with Intel Core i7-6850 CPU 3.60GHz and Nvidia Titan X Pascal GPU. The software environments were Ubuntu 16.04, Python 3.5.6, Tensorflow 1.13.1 [36], and Keras 2.3.1 [37].

### 3.2. Performance of the Proposed SC-UNET-Based Architectures

The validity of the selected connections in the UNET structure was investigated for camera-like RGB color image generation from the sparse 2D LiDAR reflection image. The three proposed architectures, such as SC-UNET w/Lv(1,2,3), w/Lv(1,2) and w/Lv(1), as shown in Figure 3c–e, were evaluated in this simulation. Two conventional networks, ED-FCN and UNET, were used for the performance comparison. To determine the performance variations with respect to the number of filters *N* in convolution layer, we conducted experiments for N= 16, 32, 48, and 64. As previously mentioned in Section 3.1, five-fold cross validation was applied in all the experiments. The PSNR, SSIM, and their corresponding standard deviations are summarized in Table 2. For the evaluation of computational complexity, the number of weights in the network and the processing time measured in millisecond per frame were analyzed. To analyze the effect of single layer connection in the proposed architecture, the simulation results for connections of SC-UNET w/Lv(1), w/Lv(2), w/Lv(3), and w/Lv(4) were also summarized. For comparison with our previous work [12], all methods were also tested on the same dataset used in [12] and the performance of asymmetric ED-FCN [12] is listed in Table 2.

As *N* increased, the PSNR and SSIM of all the architectures improved. Notably, the numbers of weights increased with respect to *N*; in other words, the computational complexity and memory requirements increased. Therefore, it was necessary to select an appropriate value of *N* according to the applications and available resources.

UNET provided better image quality performance than ED-FCN. This demonstrated that the connection between the encoder and decoder was useful, even in heterogeneous image generation. In cases of single layer connection of SC-UNET, SC-UNET w/Lv(1) showed the best performance. SC-UNET w/Lv(1) and SC-UNET w/Lv(2) outperformed UNET. This meant that connection at higher level was not appropriate. SC-UNET w/Lv(1,2,3) showed better performance than UNET. On the contrary, the proposed architectures with connections at higher level, i.e., SC-UNET w/Lv(3), w/Lv(4), and w/Lv(3,4), yielded better image quality than ED-FCN, but worse quality than UNET. SC-UNET w/Lv(1,2) outperforms all the architectures, including SC-UNET w/Lv(1,2,3). SC-UNET w/Lv(1,2) with N= 48 and 64 had better image quality performance than asymmetric ED-FCN. In particular, SC-UNET w/Lv(1,2) with N= 64 produced improvements of ‘3.87 dB in PSNR and 0.17 in SSIM’ over the asymmetric ED-FCN, respectively. These results confirmed the validity of the observations presented in Section 2.2.

As shown in Table 1, the feature map at level 1 was fully dense and the similarity between encoder and decoder feature maps was 0.821. Similarly, the sparseness and similarity at level 2 were ‘0.92% and 0.573’, respectively. Therefore, encoder feature maps at levels 1 and 2 could provide useful information for the image generation at the decoder part. In contrast, the sparseness and similarity at levels 3 and 4 were ‘8.72% and 0.567’, and ‘42.63% and 0.355’, respectively. Considering both sparseness and similarity, the encoder feature maps at levels 3 and 4 had less relevance to the decoder feature maps. This implies that the connections at levels 3 and 4 could produce undesirable influence on the image-generation performance. This explains why SC-UNET w/Lv(1,2) yielded the best performance and SC-UNET w/Lv(3,4) yielded the worst performance among other networks with connections, including UNET.

These simulation results provide the insight that the connections in SC-UNET should be selected by considering the sparseness of each level in the encoder network and the similarity between the same levels of the encoder and decoder networks.

### 3.3. Inference Examples

**Figure 4:**
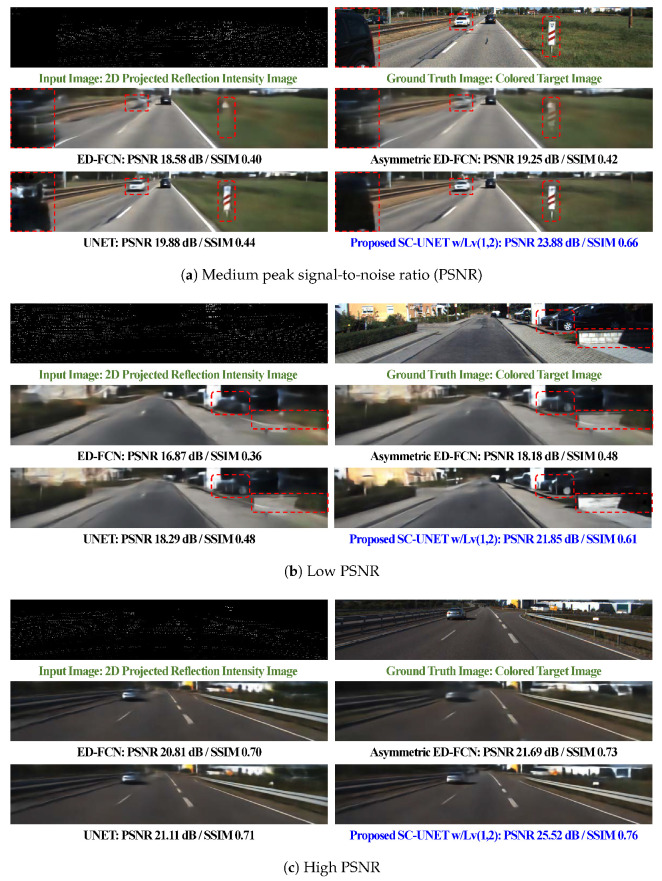
Sample inference results.

For subjective quality comparison, three inference examples for ED-FCN, UNET, asymmetric ED-FCN, and SC-UNET w/Lv(1,2) are shown in Figure 4. All three methods are tested with N=64 except symmetric ED-FCN. The examples are selected based on PSNR. The 2D LiDAR reflection and corresponding ground truth (GT) color images are also shown in the first row.

In Figure 4a, two networks without connection between encoder and decoder feature maps, such as the ED-FCN and asymmetric ED-FCN, generate very blurry objects such as white vehicle and white road-pole with red stripes. Contrarily, the UNET and SC-UNET w/Lv(1,2) produce those objects in detail. The UNET distorts short-distance black vehicle on the left side, but the proposed method faithfully generates it. Figure 4b shows that the ED-FCN does not generate tire-wheel and small wall on the right side and the proposed SC-UNET w/Lv(1,2) generates them more clearly than all others. In Figure 4c, all networks generate images with high visual quality. Similar trends mentioned above are observed. In summary, the ED-FCN and asymmetric ED-FCN generate blurry images. The proposed method faithfully generates images while the UNET produces occasionally serious distortion.

## 4. Conclusions

In this paper, we propose a SC-UNET architecture that effectively generates a camera-like RGB color image from a heterogenous sparse LiDAR reflection-intensity image. The sparseness of the encoder feature map and the similarity between the encoder and decoder feature maps are analyzed at each level of the conventional ED-FCN. At high levels, the sparseness increases and the similarity decreases. It is not reasonable to concatenate feature maps at high levels when designing a SC-UNET architecture for image generation. SC-UNET architectures with concatenation at low levels are proposed. Through simulations, we show that the proposed SC-UNET w/Lv(1,2), i.e., SC-UNET with concatenations at levels 1 and 2, outperforms the other architectures including asymmetric ED-FCN, in terms of both the objective and subjective qualities of the generated image. In particular, SC-UNET w/Lv(1,2) with N= 64 produces improvements of ‘3.87 dB in PSNR and 0.17 in SSIM’ over the asymmetric ED-FCN, respectively.

It is very important to consider the sparseness and similarity in determining the levels to be concatenated between feature maps of the encoder and decoder. The methodology is very useful in various applications where the input and output have different sparseness and heterogeneous characteristics. 

## Figures and Tables

**Table 1:** Summary of Information at Each Level of Encoder of the Conventional ED-FCN.

Level	The Number of Weights in the Encoder Feature Map	Size of Receptive Field	Sparseness (%)	Similarity
Level 4	66,304*N* (592 × 112 ×N)	5 × 5	42.63	0.355
Level 3	33,152*N* (296 × 56 × 2*N*)	18 × 18	8.72	0.567
Level 2	16,576*N* (148 × 28 × 4*N*)	52 × 52	0.92	0.573
Level 1	8288*N* (74 × 14 × 8 *N*)	136 × 136	0.00	0.821

**Table 2:** Performance results of the proposed SC-UNET-based architectures.

The Number of Filters in the First Convolution Layer	Network Architecture	The Number of Weights [ea.]	Average Processing Time [ms]	Dataset in [12]		The 5-Fold Cross Validation	
				PSNR	SSIM	PSNR [AVR. (STD.)]	SSIM [AVR. (STD.)]
N=16	ED-FCN	1,747,955	4.47	17.98	0.43	17.90 (2.12)	0.43 (0.14)
	UNET	1,943,795	4.88	18.01	0.43	17.92 (2.88)	0.43 (0.19)
	SC-UNET w/Lv(1,2,3)	1,941,491	4.53	18.04	0.44	18.01 (2.11)	0.44 (0.17)
	**SC-UNET w/Lv(1,2)**	1,932,275	4.50	**18.17**	**0.45**	**18.10 (2.91)**	**0.44 (0.15)**
	SC-UNET w/Lv(3,4)	1,759,475	4.48	17.99	0.42	17.82 (2.15)	0.41 (0.17)
	SC-UNET w/Lv(4)	1,750,259	4.48	17.99	0.43	17.90 (2.11)	0.43 (0.18)
	SC-UNET w/Lv(3)	1,757,171	4.48	18.01	0.43	17.94 (2.19)	0.43 (0.17)
	SC-UNET w/Lv(2)	1,784,819	4.48	18.08	0.44	17.99 (2.18)	0.44 (0.18)
	**SC-UNET w/Lv(1)**	1,895,411	4.49	**18.09**	**0.44**	**18.08 (2.82)**	**0.44 (0.17)**
N=32	ED-FCN	6,991,331	8.97	18.65	0.47	18.55 (2.28)	0.44 (0.11)
	UNET	7,765,475	9.49	18.88	0.49	18.81 (2.27)	0.49 (0.11)
	SC-UNET w/Lv(1,2,3)	7,018,979	9.28	18.90	0.48	18.76 (2.28)	0.46 (0.11)
	**SC-UNET w/Lv(1,2)**	7,719,395	9.34	**18.93**	**0.49**	**18.87 (2.41)**	**0.48 (0.11)**
	SC-UNET w/Lv(3,4)	6,982,115	8.87	18.69	0.48	18.56 (2.23)	0.47 (0.11)
	SC-UNET w/Lv(4)	7,028,195	9.19	18.71	0.48	18.62 (2.28)	0.47 (0.11)
	SC-UNET w/Lv(3)	7,571,939	9.31	18.88	0.49	18.76 (2.28)	0.48 (0.11)
	SC-UNET w/Lv(2)	7,129,571	9.31	18.91	0.49	18.79 (2.31)	0.48 (0.11)
	**SC-UNET w/Lv(1)**	7,756,259	9.40	**18.91**	**0.49**	**18.83 (2.39)**	**0.49 (0.11)**
N=48	ED-FCN	15,702,483	14.16	18.91	0.49	18.73 (2.20)	0.47 (0.12)
	UNET	17,465,043	15.29	19.14	0.50	19.06 (2.29)	0.50 (0.11)
	SC-UNET w/Lv(1,2,3)	17,444,307	15.12	19.98	0.55	19.88 (2.29)	0.55 (0.11)
	**SC-UNET w/Lv(1,2)**	17,361,363	15.01	**21.78**	**0.58**	**21.76 (2.49)**	**0.58 (0.11)**
	SC-UNET w/Lv(3,4)	15,806,163	14.74	19.07	0.50	18.94 (2.31)	0.49 (0.11)
	SC-UNET w/Lv(4)	15,723,219	14.34	18.99	0.49	18.86 (2.29)	0.48 (0.12)
	SC-UNET w/Lv(3)	15,785,427	14.91	19.08	0.50	18.98 (2.28)	0.49 (0.11)
	SC-UNET w/Lv(2)	16,034,259	14.95	20.12	0.55	19.96 (2.52)	0.55 (0.12)
	**SC-UNET w/Lv(1)**	17,029,587	14.96	**20.16**	**0.55**	**20.02 (2.68)**	**0.55 (0.11)**
N=64	ED-FCN	27,909,059	21.43	19.01	0.48	18.98 (2.21)	0.47 (0.11)
	UNET	31,042,499	23.13	19.37	0.52	19.27 (2.22)	0.51 (0.12)
	SC-UNET w/Lv(1,2,3)	31,005,635	22.87	20.29	0.56	19.89 (2.21)	0.56 (0.11)
	**SC-UNET w/Lv(1,2)**	30,858,179	22.71	**23.42**	**0.68**	**23.15 (2.61)**	**0.67 (0.12)**
	SC-UNET w/Lv(3,4)	28,093,379	22.30	19.16	0.51	19.08 (2.32)	0.50 (0.13)
	SC-UNET w/Lv(4)	27,945,923	21.69	19.08	0.48	18.92 (2.21)	0.48 (0.12)
	SC-UNET w/Lv(3)	28,056,515	22.56	19.31	0.51	19.16 (2.28)	0.50 (0.12)
	SC-UNET w/Lv(2)	28,498,883	22.62	21.83	0.58	21.60 (2.52)	0.58 (0.12)
	**SC-UNET w/Lv(1)**	30,268,355	22.63	**22.29**	**0.62**	**22.01 (2.65)**	**0.61 (0.12)**
-	Asymmetric ED-FCN [12]	3,350,243	7.74	19.38	0.50	19.28 (2.18)	0.50 (0.11)

**Figure A1:**
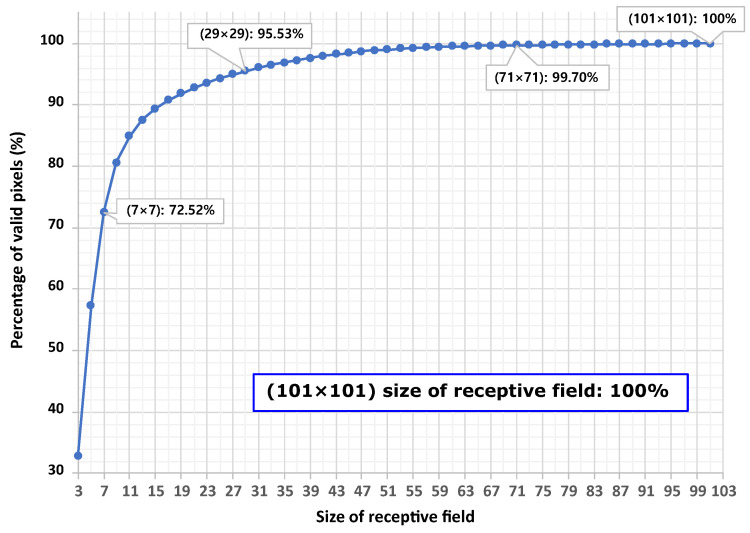
Percentage of valid pixels according to the size of the receptive field.

## Appendix A. Analysis of Sparseness Using Receptive Field

In this appendix, we provide the analysis of the relationship between sparseness and the receptive field in the ED-FCN network, as shown in Figure 2.

In general, neglecting the effect of bias in the convolution layer and the effect of the activation layer, the size of the effective receptive field is $\left[2^{P}\left(2B+1\right)\right]\times \left[2^{P}\left(2B+1\right)\right]$, where *B* and *P* represent the cumulative number of convolution blocks with a (3×3) filter and the cumulative number of pooling operations, respectively, as shown in Table A1 [38,39].

In the case of Figure 2, the size of the receptive field, $RF_{L}$ at encoder level *L* is as follows:

(A1) $$RF_{L}=\left[2^{\left(4-L\right)}\left(2\left(10-2L\right)+1\right)\right]\times \left[2^{\left(4-L\right)}\left(2\left(10-2L\right)+1\right)\right].$$

**Table A1:** Numbers of pooling and convolution operations; $P_{L}$ and $B_{L}$ denote numbers of pooling and convolution operations at level *L*, respectively; *P* and *B* denote cumulative numbers of pooling and convolution operations, respectively.

Level (*L*)	$P_{L}$	$B_{L}$	*P*	*B*
4	0	2	0	2
3	1	2	1	4
2	1	2	2	6
1	1	2	3	8
0	1	2	4	10

For example, the size of receptive field is (52 × 52) at encoder level 2. It means that if there is at least one non-zero value within a 52 × 52 square kernel centered at a certain pixel in the reflection-intensity image, the corresponding pixel in the feature map has a non-zero value by the series of convolution and pooling operations. This pixel will be called a “valid pixel” in this appendix. For evaluation, 4300 projected reflection-intensity images are used. The percentage of valid pixels for all the pixels in evaluation images are calculated with respect to the receptive field, as shown in Figure A1. For all the pixels in the feature map to be valid, the size of the receptive field should be larger than 101 × 101.

The receptive field size and sparseness at each level, according to these results, are summarized in Table A2. In the case of encoder level 2, the feature map has 99.08% valid pixels; in other words, the sparseness is 0.92%. Notably, the sparseness for the receptive field with a size of 52 × 52 is the average sparseness for 51 × 51 and 53 × 53, as size of the receptive field should be odd owing to the characteristics of the convolution operation.

**Table A2:** Size of Receptive Field and Sparseness at Each Level of Encoder.

Level (*L*)	Size of Receptive Field	Sparseness (%)
4	5 × 5	42.63
3	18 × 18	8.72
2	52 × 52	0.92
1	136 × 136	0.00
